# Crosstalk between Wnt/β-Catenin and Estrogen Receptor Signaling Synergistically Promotes Osteogenic Differentiation of Mesenchymal Progenitor Cells

**DOI:** 10.1371/journal.pone.0082436

**Published:** 2013-12-05

**Authors:** Yanhong Gao, Enyi Huang, Hongmei Zhang, Jinhua Wang, Ningning Wu, Xian Chen, Ning Wang, Sheng Wen, Guoxin Nan, Fang Deng, Zhan Liao, Di Wu, Bosi Zhang, Junhui Zhang, Rex C. Haydon, Hue H. Luu, Lewis L. Shi, Tong-Chuan He

**Affiliations:** 1 Department of Geriatrics, Xinhua Hospital of Shanghai Jiaotong University, School of Medicine, Shanghai, China; 2 Molecular Oncology Laboratory, Department of Orthopaedic Surgery and Rehabilitation Medicine, The University of Chicago Medical Center, Chicago, Illinois, United States of America; 3 Chongqing Key Laboratory for Oral Diseases and Biomedical Sciences, and the Affiliated Hospital of Stomatology, Chongqing Medical University, Chongqing, China; 4 Ministry of Education Key Laboratory of Clinical Diagnostic Medicine, and the Affiliated Hospitals of Chongqing Medical University, Chongqing, China; 5 Department of Cell Biology, and Department of Oncology of Southwest Hospital, Third Military Medical University, Chongqing, China; 6 Department of Orthopaedic Surgery, Xiang-Ya Hospital, Central South University Xiang-Ya School of Medicine, Changsha, China; Rush University Medical Center, United States of America

## Abstract

Osteogenic differentiation from mesenchymal progenitor cells (MPCs) are initiated and regulated by a cascade of signaling events. Either Wnt/β-catenin or estrogen signaling pathway has been shown to play an important role in regulating skeletal development and maintaining adult tissue homeostasis. Here, we investigate the potential crosstalk and synergy of these two signaling pathways in regulating osteogenic differentiation of MPCs. We find that the activation of estrogen receptor (ER) signaling by estradiol (E2) or exogenously expressed ERα in MPCs synergistically enhances Wnt3A-induced early and late osteogenic markers, as well as matrix mineralization. The E2 or ERα-mediated synergy can be effectively blocked by ERα antagonist tamoxifen. E2 stimulation can enhance endochondral ossification of Wnt3A-transduced mouse fetal limb explants. Furthermore, exogenously expressed ERα significantly enhances the maturity and mineralization of Wnt3A-induced subcutaneous and intramuscular ectopic bone formation. Mechanistically, we demonstrate that E2 does not exert any detectable effect on β-catenin/Tcf reporter activity. However, ERα expression is up-regulated within the first 48h in AdWnt3A-transduced MPCs, whereas ERβ expression is significantly inhibited within 24h. Moreover, the key enzyme for the biosynthesis of estrogens aromatase is modulated by Wnt3A in a biphasic manner, up-regulated at 24h but reduced after 48h. Our results demonstrate that, while ER signaling acts synergistically with Wnt3A in promoting osteogenic differentiation, Wnt3A may crosstalk with ER signaling by up-regulating ERα expression and down-regulating ERβ expression in MPCs. Thus, the signaling crosstalk and synergy between these two pathways should be further explored as a potential therapeutic approach to combating bone and skeletal disorders, such as fracture healing and osteoporosis.

## Introduction

Osteogenic differentiation and bone formation from mesenchymal progenitor cells (MPCs) are initiated and regulated by a cascade of signaling pathways. MPCs are multipotent progenitors and can be isolated from numerous tissues, but mostly from bone marrow stromal cells. MPCs can undergo self-renewal and differentiate into multiple lineages, including osteogenic, chondrogenic, and adipogenic lineages [1-3]. Osteogenic differentiation is a sequential cascade of events that recapitulates most of the skeletal development [4]. Maintaining bone homeostasis involves in bone formation and remodeling, which is regulated by numerous signaling pathways. For example, we identified BMP9 as one of the most potent BMPs among the 14 types of BMPs in inducing osteogenic differentiation of MPCs both *in vitro* and *in vivo* [5-8]. We have further found that BMP9-mediated osteogenic signaling cross-talks with several major signaling pathways, including Wnt, IGF2, retinoic acids, EGF, growth hormone, hypoxia, and MAPK pathways [9-19]. 

Wnt signaling plays a critical role in embryonic development, postnatal development, and adult tissue homeostasis [20,21]. The hallmark of canonical Wnt signaling is the stabilization of β-catenin in the cytoplasm upon activation. The canonical Wnt ligands bind to Fzd receptors and co-receptors LRP5 or LRP6, causing phosphorylation of Dvl, which inhibits GSK3β from phosphorylating β-catenin. As a result, β-catenin is stabilized and translocated into the nucleus, where β-catenin forms a transcriptional complex with Tcf/Lef to regulate the expression of target genes, such as c-myc, PPARδ, cyclin D1, and axin2 [21-24]. The role of Wnt signaling pathway in bone biology has gained considerable attention as several human pathologies of bone such as osteoporosis pseudoglioma syndrome, sclerosteosis and van Buchem’s disease have been associated with aberrant Wnt signaling [18,19,25-27]. Activating and inactivating aberrations of the canonical Wnt signaling pathway in osteogenesis result in sclerosteosis and osteoporosis, respectively [18,19,25-27]. Thus, recent studies have sought to target Wnt signaling pathway to treat osteogenic disorders. Antibodies against endogenous antagonists, such as sclerostin and Dkk-1, have shown promising results in promoting bone formation and fracture healing [18,19,26,27]. Thus, understanding the role of Wnt signaling in bone formation has not only helped elucidate the pathogenesis of bone disorders but has also led to the development of potential therapeutic avenues to treat these disorders. 

Estrogen is essential in both genders, not only for the pubertal growth spurt and skeletal maturation, but also for the accrual and maintenance of bone mass throughout adult life [28-30]. The biological actions of estrogens are mediated by estrogen binding to one of two estrogen receptors (ERs) ERα and ERβ (ERβ), which belong to the nuclear receptor superfamily [31-33]. Upon ligand binding, the ERs dimerize and bind to estrogen response elements (EREs) in the promoters of target genes [31-34]. ERs may also regulate gene expression through protein-protein interactions with other DNA-binding transcription factors in the nucleus [31-33]. The characterization of mice lacking ERα, ERβ, or both has revealed that the receptor subtypes have overlapping but distinct roles in estrogen-dependent action in vivo [31-33]. Estrogen is now considered as a major hormonal regulator of bone metabolism in both women and men [35]. The major consequence of the loss of estrogen is an increase in bone resorption although estrogen deficiency is associated with an imbalance between bone resorption and formation, suggesting that estrogen may be also important for regulating bone formation at the cellular level [35]. 

Here, we investigate the possible crosstalk and synergy between ER signaling and canonical Wnt signaling in regulating osteogenic differentiation of MPCs. Using our previously characterized mesenchymal progenitor cells iMEFs which are treated with either estradiol (E2) or adenovirus-mediated exogenous expression of ERα, we find that the activation of ER signaling synergistically enhances Wnt3A-induced both early and late osteogenic markers, as well as matrix mineralization. The E2 or ERα-mediated synergy can be effectively blocked by ERα antagonist tamoxifen. E2 stimulation on Wnt3A-transduced mouse fetal limb explants leads to an expansion of hypertrophic chondrocyte and ossification zones and an increase in mean bone density. Ectopic bone formation via subcutaneous and intramuscular injections of Wnt3A and/or ERα-transduced MPCs reveals that ERα significantly enhances the maturity and mineralization of Wnt3A-induced ectopic bone masses. Mechanistically, we demonstrate that E2 does not exert any detectable effect on Wnt/β-catenin reporter activity. However, ERα expression is up-regulated within the first 48h in AdWnt3A-transduced MPCs, whereas ERβ expression is significantly inhibited within 24h. Furthermore, the aromatase (or estrogen synthase, Cyp19) exhibits a biphasic expression pattern, up-regulated at 24h but reduced after 48h, upon Wnt3A stimulation. Thus, our results demonstrate that while ER signaling acts synergistically with Wnt3A in promoting osteogenic differentiation, Wnt3A may crosstalk with ER signaling by up-regulating ERα expression and down-regulating ERβ expression in MPCs. It is conceivable that the signaling crosstalk and synergy between these two pathways should be further explored as a potential therapeutic approach to combating bone and skeletal disorders, such as fracture healing and osteoporosis.

## Materials and Methods

### Cell culture and chemicals

HEK293 cells were obtained from ATCC (Manassas, VA, USA). Mouse mesenchymal progenitor cells iMEFs were established in our lab and previously characterized [36]. Both cell lines were maintained under conditions as described [5,9,37,38]. Chemicals 17-β-estradiol (E2) and estrogen receptor antagonist tamoxifen were purchased from Sigma-Aldrich (St. Louis, MO), and prepared in DMSO. Unless indicated otherwise, all chemicals were purchased from Fisher Scientific (Pittsburgh, PA) or Sigma-Aldrich.

### Construction and generation of recombinant adenoviruses expressing Wnt3A, ERα, RFP, and GFP

The recombinant adenoviruses were constructed by using AdEasy technology as described [5,6,39-41]. The coding regions of mouse Wnt3A and human ERα were PCR amplified and cloned into an adenoviral shuttle vector and subsequently used to generate recombinant adenoviruses in HEK293 cells. The resulting adenoviruses were designated as AdWnt3A and AdERα. AdWnt3A also expresses GFP, whereas AdERα expresses RFP as a marker for monitoring infection efficiency. Analogous adenovirus expressing only RFP (AdRFP) or GFP (AdGFP) were used as controls [9-11,22,23,39,41-43].

### Alkaline phosphatase (ALP) assay

ALP activity was assessed using a modified Great Escape SEAP Chemiluminescence Assay (BD Clontech, Mountain View, CA) and/or histochemical staining assay (using a mixture of 0.1 mg/mL of napthol AS-MX phosphate and 0.6 mg/mL of Fast Blue BB salt), as described [5,6,9-13,15,37,40,43,44]. For the chemiluminescence assays, each assay condition was performed in triplicate. The results were repeated in at least three independent experiments. ALP activity was normalized by total cellular protein concentrations among the samples.

### Mineralization assay (Alizarin Red S staining)

Alizarin Red S staining was carried out as described previously [5,6,9-11,37,40,42,43,45]. Briefly, the treated cells were cultured in the presence of ascorbic acid (50 mg/mL) and β-glycerophosphate (10mM). At 10 days after infection, mineralized matrix nodules were stained for calcium precipitation by means of alizarin red S staining. Cells were fixed with 0.05% (v/v) glutaraldehyde at room temperature for 10min. After being washed with distilled water, fixed cells were incubated with 0.4% Alizarin Red S for 5min, followed by extensive washing with distilled water. The staining of calcium mineral deposits was recorded under bright-field microscopy.

### Immunohistochemical (IHC) staining

IHC staining was carried out as previously described [13,46-49]. The cells were fixed with 10% formalin and washed with PBS, permeabilized with 1% NP-40 and blocked with 10% goat serum, followed by incubation with osteocalcin and osteopontin antibodies (Santa Cruz Biotechnology, Santa Cruz, CA) for 1h. After washing, cells were incubated with biotin-labeled secondary antibody for 30min, followed by incubating cells with streptavidin-horseradish peroxidase (HRP) conjugate for 20min. The presence of the expected proteins were visualized by diaminobenzidine (DAB) staining and examined under a microscope. Stains without the primary antibody or with control IgG were used as negative controls.

### Transfection and luciferase reporter assay

Exponentially growing cells were seeded in 25cm^2^ cell culture flasks and transfected with 2μg per flask of Tcf/β-catenin responsive luciferase reporter pTop-Luc using Lipofectamine (Invitrogen) as described [9,11,50-53]. At 16h after transfection, cells were replated to 24-well plates and treated with 10^-4^mol/L to 10^-8^mol/L estradiol. After 24h, cells were lysed, and collected for luciferase assays using Promega’s Luciferase Assay Kit (Madison, WI). Each assay condition was performed in triplicate. Luciferase activity was normalized by total cellular protein concentrations among the samples. Relative Top-Luc reporter activity was expressed as mean ± SD.

### RNA isolation and semi-quantitative RT-PCR (sqPCR) analysis

Total RNA was isolated with the TRIzol Reagents (Invitrogen) by following manufacturer’s instructions. The cDNA synthesis was carried out by reverse-transcription reaction with hexamer and M-MuLV Reverse Transcriptase (New England Biolabs, Ipswich, MA). The cDNA products were further diluted 5 to10 fold and used as PCR templates. The sqPCR was carried out as described [9,12,13,15,44,54,55]. PCR primers (Table S1) were designed by using the Primer3 program to amplify the genes of interest (approximately 150 to 180bp). A touchdown cycling program was as follows: 94°C for 2min for 1 cycle; 92°C for 20 seconds, 68°C for 30 seconds, and 72°C for 12 cycles decreasing 1°C per cycle; and then at 92°C for 20 seconds, 57°C for 30 seconds, and 72°C for 20 seconds for 20 to 25 cycles depending on the abundance of a given transcript. PCR products were resolved on 1.5% agarose gels .All samples were normalized by the expression level of GAPDH.

### Mouse fetal limb explant culture

All animal experiments reported in this study were carried out in strict accordance with the recommendations in the Guide for the Care and Use of Laboratory Animals of the National Institutes of Health. The protocol was approved by the Institutional Animal Care and Use Committee (IACUC) of The University of Chicago (protocol Number #71108). All surgery was performed under anesthesia, and all efforts were made to minimize suffering. The isolation and culture of mouse fetal limb explants was carried out as previously described [12,14,36]. Briefly, the forelimbs of mouse embryos (E18.5) were skinned, dissected under sterile conditions, and incubated in DMEM medium containing 0.5% bovine serum albumin, 50μg/mL ascorbic acid (Sigma), 1mM β-glycerophosphate, and 100μg/mL of penicillin-streptomycin at 37°C in humidified air with 5% CO2 for up to 14 days. Medium was changed every 3 days. The cultured limb explants were directly infected AdWnt3A and/or added with estradiol (10^-6^mol/L) 24h after dissection. At least five limb explants were included in each assay group. On day 7, calcein (100mM) was added to the medium. On day 10, the cultured tissues were harvested. Soft tissues were removed. The samples were subjected to fluorescence microscopy, micro-CT imaging, and histologic evaluation. 

### Micro-computed tomographic (μCT) imaging analysis

Cultured mouse fetal limb specimens were imaged using the µCT component of a GE Triumph (GE Healthcare, Piscataway, NJ) trimodality preclinical imaging system as described [12-15,36,44,56]. All image data analysis was performed using Amira 5.3 (Visage Imaging, Inc., San Diego, CA). The bone mean density heat maps were calculated as described[12,13,36,44,56].

### Stem cell implantation for ectopic bone formation

The use and care of animals was approved by the Institutional Animal Care and Use Committee. The subcutaneous and intramuscular ectopic bone formation assays were carried out as previously described [6,12-15,36,44,56]. Briefly, subconfluent iMEFs were co-infected with AdWnt3A, or AdGFP and AdERα for 24h. Infection efficiency was confirmed by fluorescence microscopy. The infected cells were collected for subcutaneous injection (5×10^6^/injection) into the flanks, or for intramuscular injection into the quadriceps of athymic nude mice (n=5 per group, 4–6-week old, male, Harlan Sprague Dawley). At 6 weeks after implantation, animals were euthanized. The implant sites were retrieved for histologic evaluation.

### Histologic evaluation

Retrieved and cultured tissues were fixed in 10% formalin, decalcified, and paraffin-embedded. Serial sections of the embedded specimens were stained with hematoxylin and eosin (H & E) as described [6,9,12,13,37,40,43,56].

### Statistical analysis

Quantitative data were expressed as mean ± SD. Statistical significances between treatment groups vs. control groups were determined by one-way analysis of variance and the two-tailed Student’s t test. A p-value of < 0.05 was defined as statistically significance.

## Results

### Activation of ER signaling enhances Wnt3A-induced early osteogenic differentiation of MPCs

**Figure 1:**
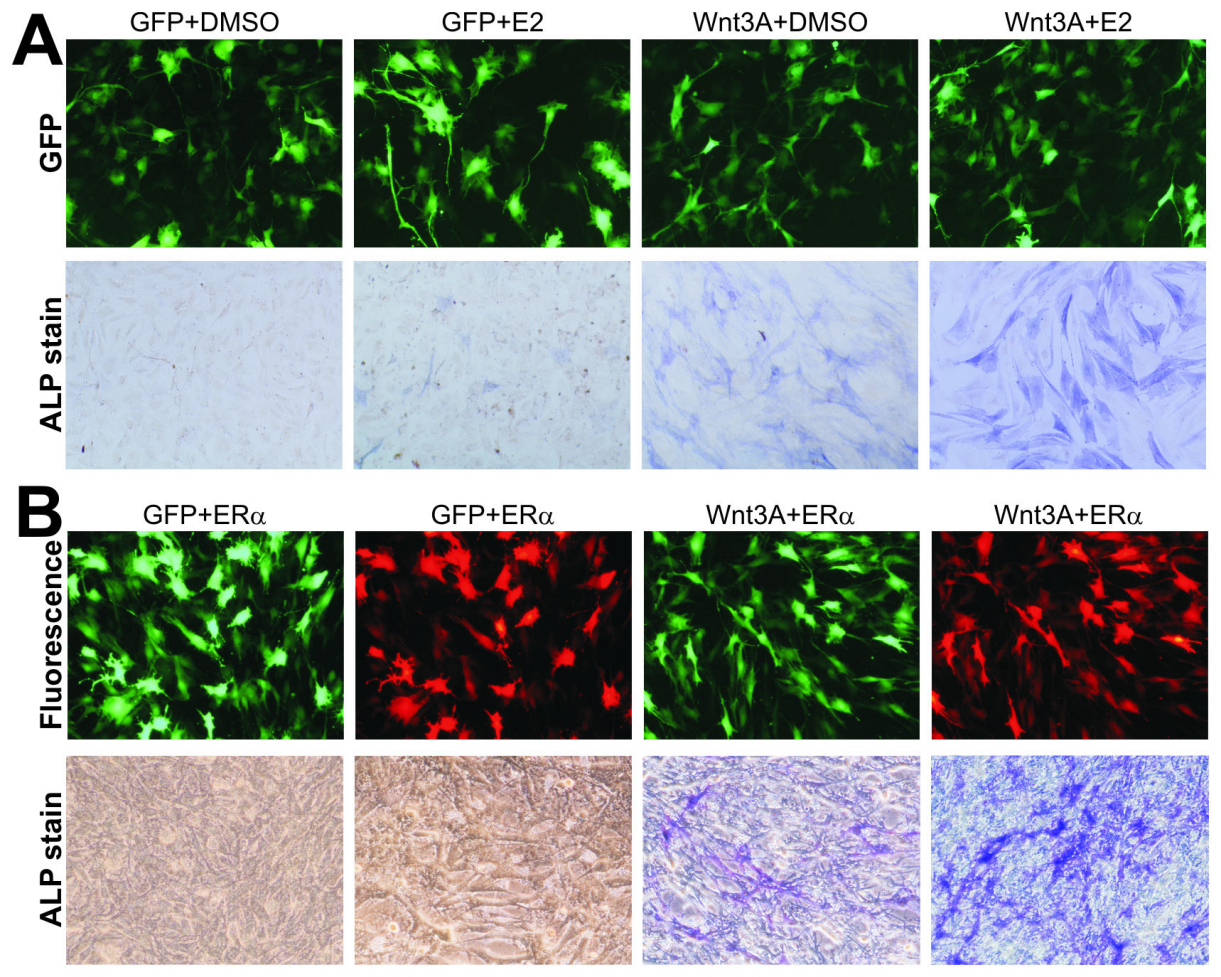
Histochemical staining of estrogen and Wnt3A-stimulated ALP activity in MPCs. (**A**) Subconfluent iMEFs were infected with AdWnt3A or AdGFP and treated with estradiol (E2, 0.1μM) or DMSO. Adenovirus infection efficiency was documented at 24h post infection (top panel). At 5 days after infection/treatment, cells were fixed and subjected to ALP histochemical staining. Representative results are shown. (**B**) ALP activity in MPCs co-expressing Wnt3A and estrogen receptor α (ERα). Subconfluent iMEFs were co-infected with AdWnt3A and AdERα or AdGFP. Transduction efficiency was monitored for GFP (AdWnt3A and AdGFP) or RFP (AdERα) at 24h. Cells were fixed and stained for ALP activity at day 5. Representative results are shown.

To determine if estrogen receptor signaling pathway exerts any effect on Wnt3A-induced osteogenic differentiation, we tested the effect of estradiol (E2) on Wnt3A-stimulated MPC line iMEFs. Exogenous expression of Wnt3A was mediated by a recombinant adenoviral vector, which was shown to transduce iMEFs with high efficiency (Figure **1A**). As previously reported [9], Wnt3A was shown to induce early osteogenic marker ALP activity, which was further enhanced by the presence of E2 (0.1μM) (Figure **1A**, bottom row) although E2 alone induced negligible ALP activity. To further demonstrate that these results were caused by the activation of estrogen receptor signaling pathway, we constructed an adenoviral vector to expresses human ERα, namely AdERα, which co-expresses RFP as a marker. The AdERα-mediated expression of human ERα was confirmed by sqPCR and Western blot (data not shown). When iMEFs were co-infected with Wnt3A and ERα adenoviruses, there was a significant increase in ALP activity in MPCs when compared with the Wnt3A alone group, although ERα overexpression alone exerted minimal or undetectable effect (Figure **1B**). 

**Figure 2:**
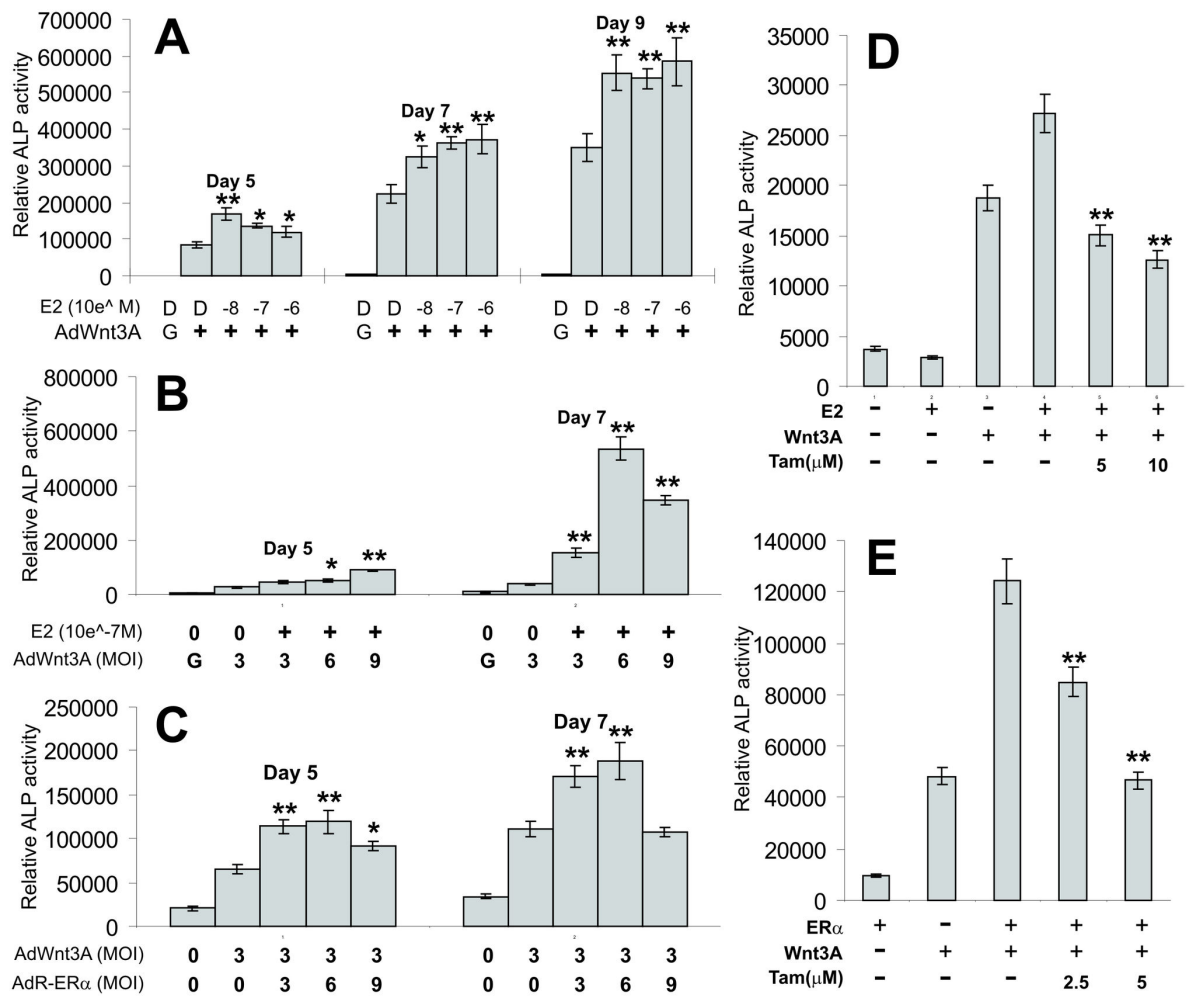
Estrogen-mediated synergistic effect on Wnt3A-stimulated ALP activity can be blocked by tamoxifen. (**A**) E2 dose-dependent synergy with Wnt3A in MPCs. Subconfluent iMEFs were infected with a fixed titer (MOI=5) of AdWnt3A or AdGFP, and treated with various concentrations of estradiol or DMSO. ALP activity was determinedusing a modified Great Escape SEAP chemiluminescence assay at the indicated time points (see Methods). D, DMSO solvent control; G, AdGFP control. (**B**) Wnt3A dose-dependent synergy with E2 in MPCs. Subconfluent iMEFs were infected with varied titers of AdWnt3A or AdGFP, and treated with estradiol (0.1μM) or DMSO. ALP activity was determined at the indicated time points. (**C**) ERα expression synergizes with Wnt3A in MPCs. The iMEFs were co-infected with AdWnt3A (MOI=3) or AdGFP (MOI=3) and varied titers of AdERα. ALP activity was determined at the indicated time points. (**D**) E2-induced synergy can be blocked by tamoxifen. The iMEFs were infected with AdWnt3A or AdGFP (MOI=3), and treated with estradiol (0.1μM) or DMSO in the presence or absence of tamoxifen (0 to 10μM). ALP activity was determined at day 5. (**E**) ERα-induced synergy can be blocked by tamoxifen. The iMEFs were co-infected with AdWnt3A or AdGFP and AdERα (MOI=3 each) in the presence or absence of tamoxifen (2.5 and 5μM). ALP activity was determined at day 5. All assay conditions were done in triplicate. “*”, *p*<0.05; “**”, *p*<0.01.

We further analyzed that dose-dependent nature of E2 or ERα on Wnt3A-induced ALP activity in MPCs, vice versa. When iMEF cells were transduced with a fixed titer of AdWnt3A, E2 was shown to enhance ALP activity in a dose-dependent manner and plateaued at around 10^-8^M (Figure **2A**), compared with that of the DMSO controls (at least *p<0.05*). Accordingly, when iMEFs were stimulated with E2 (10^-7^M), transduction with increasing titers of AdWnt3A led to a dose-dependent increase of ALP activity at day 5 and day 7 (except the highest titer) (at least *p<0.05*) (Figure **2B**). We obtained slimier dose-dependent increases of ALP activity when escalating titers of AdERα were used to co-infect iMEFs with AdWnt3A (Figure **2C**), although we did observe a drop in ALP activity at the highest virus titer, which might have been caused by virus toxicity. Furthermore, we examined if the synergistic effect exerted by E2 and ERα could be reversed by tamoxifen. We found that E2-enhanced ALP activity in Wnt3A-stimulated iMEFs was significantly inhibited by tamoxifen at 5μM (*p<0.05*) and 10μM (*p<0.01*) (Figure **2D**). Similarly, the synergistic effect between Wnt3A and ERα on ALP activity was effectively inhibited by tamoxifen at 2.5μM (*p<0.05*) and 5μM (p<0.01) (Figure **2E**). Taken together, these results suggest that E2 stimulation and exogenous ERα expression may enhance Wn3A-induced osteogenic differentiation. 

### Activation of ER signaling pathway synergizes with Wnt3A-induced late stage of osteogenic differentiation in MPCs

**Figure 3:**
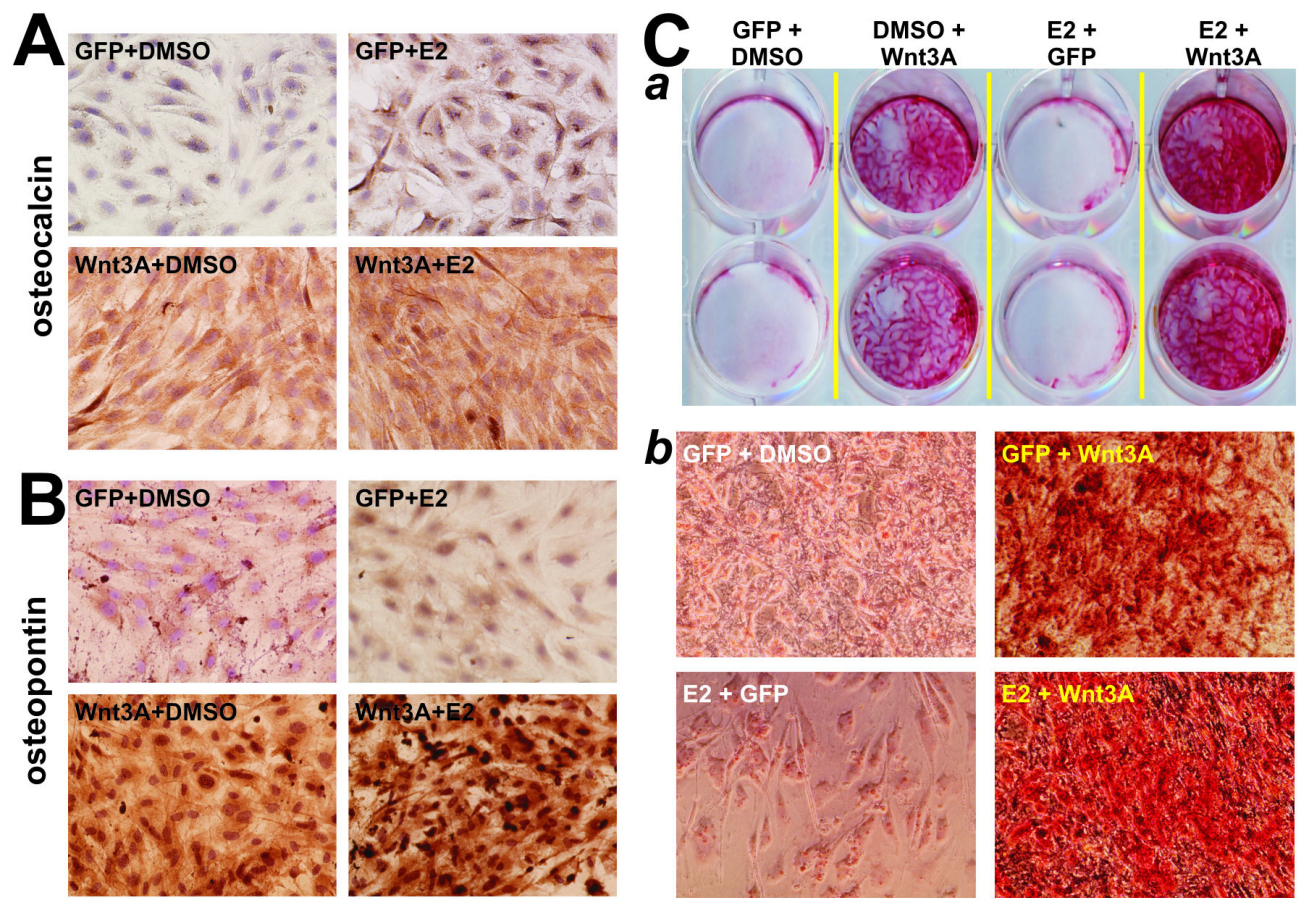
Activation of estrogen signaling synergizes with Wnt3A-induced late stage of osteogenic differentiation. (**A**) & (**B**) Estradiol synergizes with Wnt3A in induction of late osteogenic markers osteocalcin and osteopontin expression. Subconfluent iMEFs were infected with AdWnt3A (MOI=5) or AdGFP, and treated with estradiol (0.1μM) or DMSO. At day 10, cells were fixed and subjected to immunohistochemical staining with antibodies against osteocalcin (**A**) or osteopontin (**B**). Isotype IgG or no primary antibodies were used as negative controls (data not shown). Representative results are shown. (**C**) E2 enhances Wnt3A-induced matrix mineralization. The iMEF cells were treated as described in (**A**) and maintained in mineralization medium. At day 14, cells were fixed and subjected to Alizarin Red S staining. Macrographic images (*a*) and microscopic images (*b*) were recorded. Representative results are shown.

We analyzed the effect of E2 stimulation and exogenous ERα expression on Wnt3A-induced late osteogenic marker and matrix mineralization. When Wnt3A-transduced iMEFs were treated 10^-7^M E2, the expression of late osteogenic markers osteocalcin (Figure **3A**) and osteopontin (Figure **3B**) was increased when compared with the Wnt3A alone groups, while E2 alone did not exert any significant effect. Similar results were also obtained when AdERα was used in the place of E2 stimulation (data not shown). 

We also analyzed the synergistic effect of E2 and Wnt3A on matrix mineralization, and found that E2 effectively enhanced Wnt3A-induced mineral nodule formation compared with the Wnt3A alone group (Figure **3C**). Consistently with the findings from ALP studies, E2 stimulation alone did not result in any significant mineral nodule formation. These results further indicate that E2 stimulation could significantly enhanced Wnt3A-induced terminal osteogenic differentiation of MPCs. 

### Estradiol treatment enhances the mean bone density and endochondral ossification in Wnt3A-treated mouse fetal limb explants

**Figure 4:**
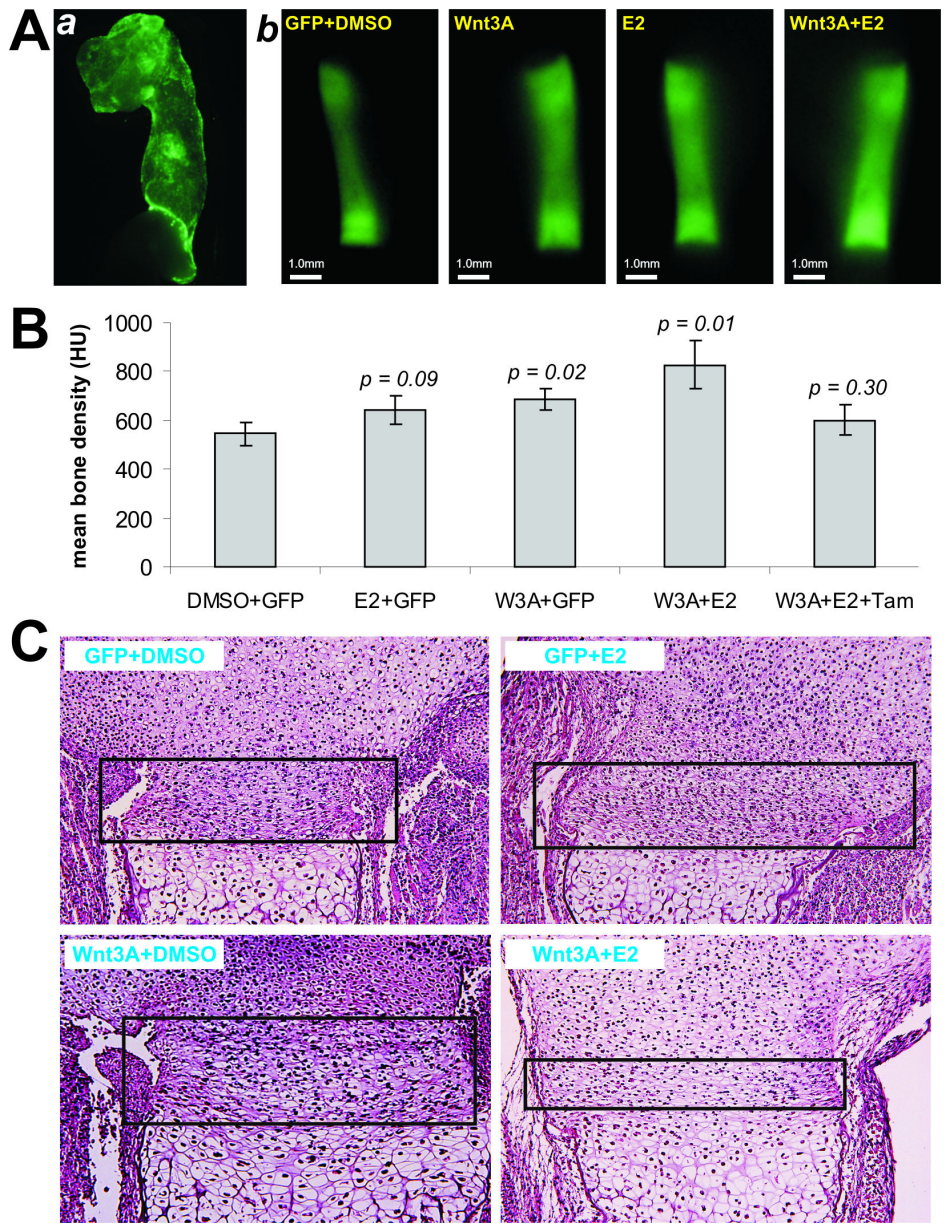
Estradiol enhances the bone density in Wnt3A-treated mouse fetal limb explants. (**A**) Mouse fetal limb explant culture and Wnt3A gene transfer. The skinned forelimbs of mouse embryos (E18.5) were dissected, and directly infected AdWnt3A, or AdGFP (**a**). At 24h after dissection, the limb explants were treated with estradiol (10^-6^M) or DMSO (n=5 each group). At day 7, calcein (100mM) was added to the medium. The cultured tissues were harvested on day 10 and soft tissues were removed (**b**). (**B**) The treated limb explants were subjected to fixed and subjected to μCT imaging. Mean bone density of was calculated. (**C**) H & E staining of the cultured limb explants. The cultured limb samples were decalcified, paraffin-embedded and subjected to H & E staining. The growth plate was indicated with boxes. Representative images are shown.

To further test the synergistic effect of E2 and Wnt3A on bone formation, we used an ex vivo mouse fetal limb culture model. We and others have used such model to demonstrate the biological effects of several important osteogenic factors on bone formation and endochondral ossification [12,14,36]. The dissected mouse fetal forelimbs were effectively transduced with AdWnt3A (Figure **4A** **, panel a**). Using fluorescence labeling dye calcein, we were able to trace the new bone formation (Figure **4** **, panel b**). At the endpoint of assays the limb cultures were fixed and subjected to micro-CT scanning. We found that both Wnt3A and Wnt3A+E2 groups exhibited significantly higher mean bone density than that of the GFP+DMSO control (p<0.02 and p<0.01, respectively) (Figure **4B**). There was a trend that Wnt3A+E2 group had higher mean bone density than that of Wnt3A alone group although the difference was not statistically significant (p=0.06). Furthermore, the addition of tamoxifen nullified the significance between Wnt3A+E2 group and GFP+DMSO control group (Figure **4B**). It is noteworthy that the GFP+E2 group exhibits higher mean bone density than that of the DMSO+GFP group’s although the difference is not statistically significant (p=0.09). Histologic analysis revealed that the resting and proliferating zones were reduced while the hypertrophic zone and ossification zone were expanded in Wnt3A+E2 treatment group (Figure **4C**). Wnt3A group exhibits an expanded hypertrophic zone, and E2 alone did not significantly affect the growth plate (Figure **4C**). These results suggest that the synergistic effect of Wnt3A and E2 may lead to accelerated endochondral ossification in the fetal limb culture model. 

### Exogenous expression of ERα and Wnt3A synergistically induces ectopic bone formation

**Figure 5:**
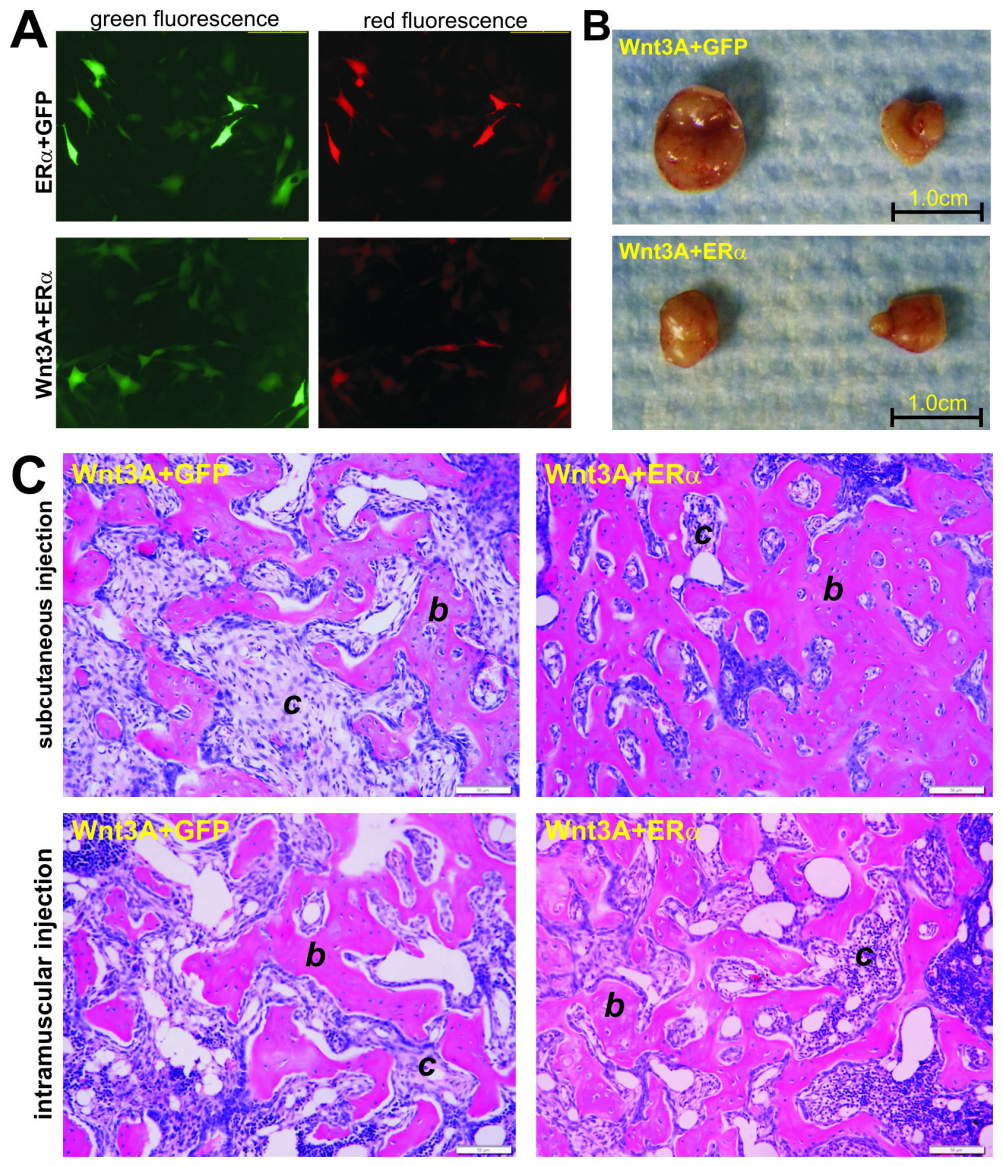
ERα and Wnt3A synergistically induce ectopic bone formation. (**A**) Co-expression of Wnt3A and ERα in MPCs. Subconfluent iMEFs were co-infected with AdWnt3A and AdERα, or AdGFP. Fluorescence signal was examined at 24h post infection. (**B**) The transduced cells described in (**A**) were collected and injected into athymic nude mice subcutaneously and intramuscularly. Ectopic bony masses were harvested after 6 weeks. The GFP only and ERα+GFP group did not form any masses during the experimental period. Representative results for subcutaneous masses are shown. (**C**) H & E staining of the ectopic bony masses. The retrieved masses were decalcified, paraffin-embedded and subjected to H & E staining. Representative images are shown. “b”, osteoid matrix; “c”, injected/undifferentiated cells.

We next tested the synergistic effect of ERα and Wnt3A on ectopic bone formation *in vivo*. The iMEFs were first transduced with AdWnt3A and AdERα, or AdGFP (Figure **5A**), and the transduced cells were used for either subcutaneous injections or intramuscular injections of aythmic nude mice. Bony masses were found in the Wnt3A+GFP group and Wnt3A+ERα group, but not in GFP only or ERα+GFP group (Figure **5B**). The retrieved masses were decalcified, paraffin-embedded and subjected to H & E staining. We found that in both subcutaneous and intramuscular injections, Wnt3A+ERα group formed more mature and thicker trabecular bone matrices than that of the Wnt3A alone group (Figure **5C**). These in vivo results are consistent with the above-mentioned *in vitro* and *ex vivo* findings, and strongly suggest that activation of estrogen receptor α signaling may exhibit strong synergistic effect on canonical Wnt-induced osteogenic differentiation of MPCs although ERα signaling per se may not be sufficient to induce robust osteogenic differentiation of MPCs. 

### Wnt3A may crosstalk with ER signaling pathway by up-regulating ERα expression at the early stage in MPCs

**Figure 6:**
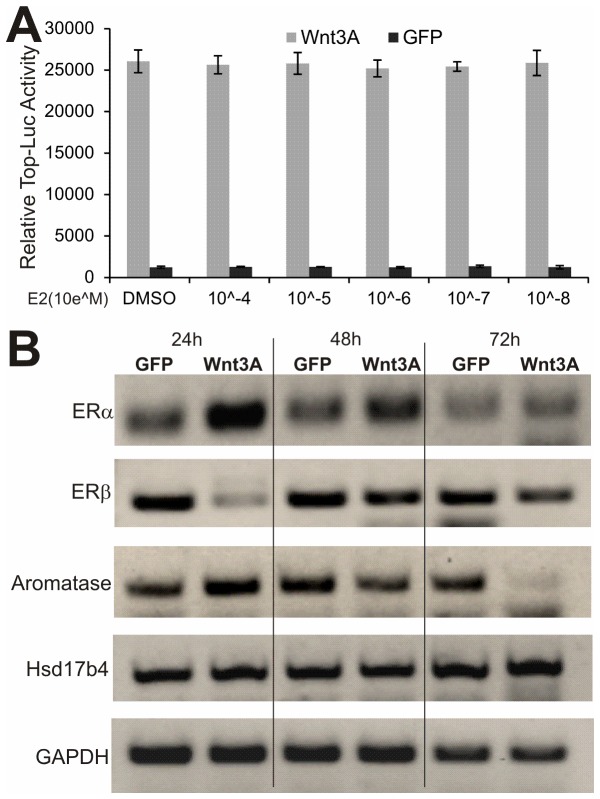
Wnt3A upregulates ERα, but not ERβ, expression at the early stage in MPCs. (**A**) Estradiol does not affect Wnt/β-catenin signaling activity. Subconfluent iMEFs were transfected with the Tcf/β-catenin reporter TOP-Luc, and infected with AdWnt3A or AdGFP, and then treated with varied concentrations of estradiol. Luciferase activity was measured at 48h post treatment. Each assay condition was done in triplicate. (**B**) Wnt3A upregulates ERα expression in MPCs. Subconfluent iMEFs were infected with AdWnt3A or AdGFP. Total RNA was collected at 24h, 48h, and 72h after infection and subjected to RT-PCR analysis using primers specific for mouse ERα, ERβ, Aromatase, and Hsd17b4. All samples were normalized with GAPDH expression level. Representative results are sown.

To explore the possible mechanistic underpinning of the synergistic effect between ERα and Wnt3A on osteogenic differentiation, we analyzed if E2 stimulation would affect Wnt3A signaling activity, or vice versa. Utilizing the commonly-used Tcf/β-catenin reporter pTop-Luc, we tested a broad range of concentrations of E2 for its effect on pTop-Luc reporter activity. As shown in Figure 6**A**, E2 stimulation did not significantly affect the reporter activity, and furthermore, E2 stimulation did not act synergistically on Wnt3A-induced reporter activity, suggesting that estrogen signaling may not act as an upstream regulator of canonical Wnt pathway in MPCs. 

We further tested if Wnt signaling regulates ERα pathway in MPCs. We transduced iMEFs with AdWnt3A or AdGFP for 24h, 48h and 72h. The expression of ERα, ERβ, aromatase, and Hsd17b4 was determined by semi-quantitative RT-PCR. We found that ERα expression was up-regulated by Wnt3A at 24h and 48h, but returned to baseline at 72h (Figure **6B**). Interestingly, the expression of ERβ was significantly down-regulated at 24h upon Wnt3A stimulation, and gradually returned to base level after 48h (Figure **6B**). The expression of aromatase (also known as estrogen synthase or CYP19), an enzyme responsible for a key step in the biosynthesis of estrogens, was biphasic, up-regulated at 24h but reduced after 48h, upon Wnt3A stimulation, indicating that there may be a feedback inhibition mechanism (Figure **6B**). Lastly, we examined the expression of Hsd17b4 gene that encodes an enzyme involved in peroxisomal fatty acid beta-oxidation and catalyzes the oxidation of estradiol with high preference over the reduction of estrone [57]. We found that Hsd17b4 was slightly up-regulated at later time point (72h) (Figure **6B**), suggesting Hsd17b4 expression may not be directly regulated by Wnt3A. Although the detailed mechanism underlying the ERα synergistic effect on Wnt3A-induced osteogenic differentiation remains to be fully elucidated, our findings strongly suggest that the signaling crosstalk and synergy of the two pathways should be further explored as potential novel approach to combating bone and skeletal disorders, such as osteoporosis.

## Discussion

Both estrogen and canonical Wnt signaling pathways play an important role in regulating bone development and bone hemostasis. Here we investigate the possible crosstalk and synergy of the two pathways in regulating osteogenic differentiation of MPCs. Through either the administration of E2 or exogenous expression of ERα in MPCs, we find that the activation of estrogen receptor signaling synergistically enhances Wnt3A-induced both early and late osteogenic markers, as well as matrix mineralization. The E2 or ERα-mediated synergy can be effectively blocked by ERα antagonist tamoxifen. E2 stimulation on Wnt3A-transduced mouse fetal limb explants leads to an expansion of hypertrophic chondrocyte zone and ossification and an increase in mean bone density. Ectopic bone formation via subcutaneous and intramuscular injections of Wnt3A and/or ERα-transduced MPCs reveals that ERα significantly enhances the maturity and mineralization of Wnt3A-induced ectopic bone masses, compared with Wnt3A treatment alone. Mechanistically, we demonstrate that E2 does not exert any detectable effect on Wnt/β-catenin reporter activity. However, ERα expression is up-regulated within the first 48h in AdWnt3A-transduced MPCs, whereas ERβ expression is significantly inhibited within 24h. Furthermore, the aromatase (also known as estrogen synthase, or Cyp19), a key enzyme for the biosynthesis of estrogens, exhibits a biphasic expression pattern, up-regulated at 24h but reduced after 48h, upon Wnt3A stimulation. Thus, our results demonstrate that estrogen signaling acts synergistically with Wnt3A in promoting osteogenic differentiation and suggest that Wnt3A may crosstalk with estrogen signaling by up-regulating ERα expression and down-regulating ERβ expression in MPCs.

Our findings indicate that Wnt3A can regulate the expression of ERα and ERβ in an opposite fashion, which may be consistent with numerous reports about the antagonist relationship between the two receptors [28-30]. The biological actions of estrogens are mediated by estrogen binding to ERα or ERβ. Mice lacking ERα, or ERβ, or both has revealed that both receptor subtypes have overlapping but distinct functions in estrogen-dependent action in vivo [31-33]. ERα and ERβ have different transcriptional activities under different ligand, cell-type, and promoter contexts. Both receptors can form functional heterodimers although the biological roles of ERα /β heterodimers are unknown. When co-expressed, ERβ inhibits ERα-mediated gene expression and in many instances opposes the actions of ERα [31-33], although the role of ERα in osteoblast lineage cells has remained elusive. A recent study indicated that ERα in osteoblast progenitors expressing Osterix1 (Osx1) potentiates Wnt/β-catenin signaling, leading to an increase in proliferation and differentiation of periosteal cells and the optimal cortical bone accrual at the periosteum in mice [58]. However, this ERα function does not require estrogens. 

It was reported that ERα may directly interact with β-catenin in human colon and breast cancer cells [59]. However, it may require further investigation about the direct feature of the interaction because a polycomb group protein EZH2 was also shown to interact directly with both ERα and β-catenin, thus connecting the estrogen and Wnt signaling circuitries in breast and prostate cancer cells [60]. It remains to be determined if ERα and β-catenin directly interact each other in MPCs and osteoblast progenitor cells. Nonetheless, it was reported that, in osteoblastic ROS 17/2.8 cells and primary osteoblast cells, Wnt/β-catenin signaling was a component of osteoblastic cell early responses to load-bearing and its effectiveness required ERα [61]. In MC3T3-E1 osteoblastic cells, estrogen receptor and Wnt signaling were shown to interact to regulate early gene expression in response to mechanical strain [62]. It was shown that E2 sensitized the effect on mechanically induced Cox-2 expression, which could be abolished using the anti-estrogen ICI182780. However, mechanical strain in the presence of Wnt signaling activators diminished both the E2 sensitizing effect and the stimulatory effect of Wnt signaling in the absence of strain [62]. A more recent study focused on the role of ERα in osteoblast lineage cells by deleting ERα at different stages of differentiation in murine osteoblast lineage cells [58]. It was found that ERα in osterix1-positive osteoblast progenitors potentiates Wnt/β-catenin signaling, thereby increasing proliferation and differentiation of periosteal cells although this function did not require estrogens [58]. Thus, the molecular mechanism underlying the interplay between estrogen signaling and Wnt/β-catenin pathway remains to be thoroughly elucidated. 

In summary, we investigate the possible crosstalk and synergy between ER signaling and canonical Wnt signaling in regulating osteogenic differentiation of MPCs. Our results demonstrate that the activation of ER signaling via estradiol and exogenously expressed ERα in MPCs synergistically enhances Wnt3A-induced both early and late osteogenic markers, as well as matrix mineralization. The E2 or ERα-mediated synergy can be effectively blocked by tamoxifen. E2 stimulation enhances endochondral ossification of Wnt3A-transduced mouse fetal limb explants. Exogenously expressed ERα in MPCs significantly enhances the maturity and mineralization of Wnt3A-induced ectopic bone masses. Mechanistically, we show that Wnt3A up-regulates the expression of ERα and aromatase but down-regulates ERβ expression in MPCs. It is conceivable that the signaling crosstalk and synergy between the ER signaling and Wnt/β-catenin pathways may be further explored as potential novel approach to combating bone and skeletal disorders, such as osteoporosis.

## Supporting Information

Table S1 **Oligonucleotides for cloning and RT-PCR.**
(XLS)

## References

[1] Prockop DJ (1997) Marrow stromal cells as stem cells for nonhematopoietic tissues. Science 276: 71-74. doi:10.1126/science.276.5309.71. PubMed: 9082988. 9082988

[2] Pittenger MF, Mackay AM, Beck SC, Jaiswal RK, Douglas R et al. (1999) Multilineage potential of adult human mesenchymal stem cells. Science 284: 143-147. doi:10.1126/science.284.5411.143. PubMed: 10102814. 10102814

[3] Deng ZL, Sharff KA, Tang N, Song WX, Luo J et al. (2008) Regulation of osteogenic differentiation during skeletal development. Front Biosci 13: 2001-2021. doi:10.2741/2819. PubMed: 17981687. 17981687

[4] Olsen BR, Reginato AM, Wang W (2000) Bone development. Annu Rev Cell Dev Biol 16: 191-220. doi:10.1146/annurev.cellbio.16.1.191. PubMed: 11031235. 11031235

[5] Cheng H, Jiang W, Phillips FM, Haydon RC, Peng Y et al. (2003) Osteogenic activity of the fourteen types of human bone morphogenetic proteins (BMPs). J Bone Joint Surg Am 85-A: 1544-1552. PubMed: 12925636. 12925636 10.2106/00004623-200308000-00017

[6] Kang Q, Sun MH, Cheng H, Peng Y, Montag AG et al. (2004) Characterization of the distinct orthotopic bone-forming activity of 14 BMPs using recombinant adenovirus-mediated gene delivery. Gene Ther 11: 1312-1320. doi:10.1038/sj.gt.3302298. PubMed: 15269709. 15269709

[7] Luu HH, Song WX, Luo X, Manning D, Luo J et al. (2007) Distinct roles of bone morphogenetic proteins in osteogenic differentiation of mesenchymal stem cells. J Orthop Res 25: 665-677. doi:10.1002/jor.20359. PubMed: 17290432. 17290432

[8] Luther G, Wagner ER, Zhu G, Kang Q, Luo Q et al. (2011) BMP-9 Induced Osteogenic Differentiation of Mesenchymal Stem Cells: Molecular Mechanism and Therapeutic Potentia. Curr Gene Ther 11: 229-240. doi:10.2174/156652311795684777. PubMed: 21453282. 21453282

[9] Tang N, Song WX, Luo J, Luo X, Chen J et al. (2009) BMP9-induced osteogenic differentiation of mesenchymal progenitors requires functional canonical Wnt/beta-catenin signaling. J Cell Mol Med 13: 2448-2464. doi:10.1111/j.1582-4934.2008.00569.x. PubMed: 19175684. 19175684 PMC4940786

[10] Si W, Kang Q, Luu HH, Park JK, Luo Q et al. (2006) CCN1/Cyr61 Is Regulated by the Canonical Wnt Signal and Plays an Important Role in Wnt3A-Induced Osteoblast Differentiation of Mesenchymal. Stem Cells - Mol Cell Biol 26: 2955-2964. 16581771 10.1128/MCB.26.8.2955-2964.2006 PMC1446962

[11] Luo Q, Kang Q, Si W, Jiang W, Park JK et al. (2004) Connective Tissue Growth Factor (CTGF) Is Regulated by Wnt and Bone Morphogenetic Proteins Signaling in Osteoblast Differentiation of Mesenchymal. Stem Cells - J Biol Chem 279: 55958-55968. 15496414 10.1074/jbc.M407810200

[12] Chen L, Jiang W, Huang J, He BC, Zuo GW et al. (2010) Insulin-like growth factor 2 (IGF-2) potentiates BMP-9-induced osteogenic differentiation and bone formation. J Bone Miner Res 25: 2447-2459. doi:10.1002/jbmr.133. PubMed: 20499340. 20499340 PMC3179288

[13] Zhang W, Deng ZL, Chen L, Zuo GW, Luo Q et al. (2010) Retinoic acids potentiate BMP9-induced osteogenic differentiation of mesenchymal progenitor cells. PLOS ONE 5: e11917. doi:10.1371/journal.pone.0011917. PubMed: 20689834. 20689834 PMC2912873

[14] Liu X, Qin J, Luo Q, Bi Y, Zhu G et al. (2013) Cross-talk between EGF and BMP9 signalling pathways regulates the osteogenic differentiation of mesenchymal stem cells. J Cell Mol Med, 17: 1160–72. PubMed: 23844832. 23844832 10.1111/jcmm.12097 PMC4118175

[15] Hu N, Jiang D, Huang E, Liu X, Li R et al. (2013) BMP9-regulated angiogenic signaling plays an important role in the osteogenic differentiation of mesenchymal progenitor cells. J Cell Sci, 126: 532–41. PubMed: 23203800. 23203800 10.1242/jcs.114231 PMC3613181

[16] Xu DJ, Zhao YZ, Wang J, He JW, Weng YG et al. (2012) Smads, p38 and ERK1/2 are involved in BMP9-induced osteogenic differentiation of C3H10T1/2 mesenchymal stem cells. BMB Rep 45: 247-252. doi:10.5483/BMBRep.2012.45.4.247. PubMed: 22531136. 22531136

[17] Zhao Y, Song T, Wang W, Wang J, He J et al. (2012) P38 and ERK1/2 MAPKs act in opposition to regulate BMP9-induced osteogenic differentiation of mesenchymal progenitor cells. PLOS ONE 7: e43383. doi:10.1371/journal.pone.0043383. PubMed: 22912865. 22912865 PMC3422272

[18] Wagner ER, Zhu G, Zhang BQ, Luo Q, Shi Q et al. (2011) The therapeutic potential of the Wnt signaling pathway in bone disorders. Curr Mol Pharmacol 4: 14-25. doi:10.2174/1874-470211104010014. PubMed: 20825362. 20825362

[19] Kim JH, Liu X, Wang J, Chen X, Zhang H et al. (2013) Wnt signaling in bone formation and its therapeutic potential for bone diseases. Ther Adv Musculoskelet. Drosophila Inf Service 5: 13-31. 10.1177/1759720X12466608 PMC3582304 23514963

[20] Nusse R, Varmus HE (1982) Many tumors induced by the mouse mammary tumor virus contain a provirus integrated in the same region of the host genome. Cell 31: 99-109. doi:10.1016/0092-8674(82)90409-3. PubMed: 6297757. 6297757

[21] Clevers H, Nusse R (2012) Wnt/beta-catenin signaling and disease. Cell 149: 1192-1205. doi:10.1016/j.cell.2012.05.012. PubMed: 22682243. 22682243

[22] He TC, Chan TA, Vogelstein B, Kinzler KW (1999) PPARdelta is an APC-regulated target of nonsteroidal anti-inflammatory drugs. Cell 99: 335-345. doi:10.1016/S0092-8674(00)81664-5. PubMed: 10555149. 10555149 PMC3779681

[23] He TC, Sparks AB, Rago C, Hermeking H, Zawel L et al. (1998) Identification of c-MYC as a target of the APC pathway [see comments]. Science 281: 1509-1512. doi:10.1126/science.281.5382.1509. PubMed: 9727977. 9727977

[24] Tetsu O, McCormick F (1999) Beta-catenin regulates expression of cyclin D1 in colon carcinoma cells. Nature 398: 422-426. doi:10.1038/18884. PubMed: 10201372. 10201372

[25] Baron R, Rawadi G (2007) Wnt signaling and the regulation of bone mass. Curr Osteoporos Rep 5: 73-80. doi:10.1007/s11914-007-0006-0. PubMed: 17521509. 17521509

[26] Monroe DG, McGee-Lawrence ME, Oursler MJ, Westendorf JJ (2012) Update on Wnt signaling in bone cell biology and bone disease. Gene 492: 1-18. doi:10.1016/j.gene.2011.10.044. PubMed: 22079544. 22079544 PMC3392173

[27] Williams BO, Insogna KL (2009) Where Wnts went: the exploding field of Lrp5 and Lrp6 signaling in bone. J Bone Miner Res 24: 171-178. doi:10.1359/jbmr.081235. PubMed: 19072724. 19072724 PMC3276354

[28] Grumbach MM, Auchus RJ (1999) Estrogen: consequences and implications of human mutations in synthesis and action. J Clin Endocrinol Metab 84: 4677-4694. doi:10.1210/jc.84.12.4677. PubMed: 10599737. 10599737

[29] Juul A (2001) The effects of oestrogens on linear bone growth. Hum Reprod Update 7: 303-313. doi:10.1093/humupd/7.3.303. PubMed: 11392377. 11392377

[30] Simm PJ, Bajpai A, Russo VC, Werther GA (2008) Estrogens and growth. Pediatr Endocrinol Rev 6: 32-41. PubMed: 18806723. 18806723

[31] Björnström L, Sjöberg M (2005) Mechanisms of estrogen receptor signaling: convergence of genomic and nongenomic actions on target genes. Mol Endocrinol 19: 833-842. doi:10.1210/me.2004-0486. PubMed: 15695368. 15695368

[32] Burns KA, Korach KS (2012) Estrogen receptors and human disease: an update. Arch Toxicol 86: 1491-1504. doi:10.1007/s00204-012-0868-5. PubMed: 22648069. 22648069 PMC4782145

[33] Cheskis BJ, Greger JG, Nagpal S, Freedman LP (2007) Signaling by estrogens. J Cell Physiol 213: 610-617. doi:10.1002/jcp.21253. PubMed: 17886255. 17886255

[34] Matthews J, Gustafsson JA (2003) Estrogen signaling: a subtle balance between ER alpha and ER beta. Mol Interv 3: 281-292. doi:10.1124/mi.3.5.281. PubMed: 14993442. 14993442

[35] Khosla S, Oursler MJ, Monroe DG (2012) Estrogen and the skeleton. Trends Endocrinol Metab 23: 576-581. doi:10.1016/j.tem.2012.03.008. PubMed: 22595550. 22595550 PMC3424385

[36] Huang E, Bi Y, Jiang W, Luo X, Yang K et al. (2012) Conditionally Immortalized Mouse Embryonic Fibroblasts Retain Proliferative Activity without Compromising Multipotent Differentiation Potential. PLOS ONE 7: e32428. doi:10.1371/journal.pone.0032428. PubMed: 22384246. 22384246 PMC3285668

[37] Luo X, Chen J, Song WX, Tang N, Luo J et al. (2008) Osteogenic BMPs promote tumor growth of human osteosarcomas that harbor differentiation defects. Lab Invest 88: 1264-1277. doi:10.1038/labinvest.2008.98. PubMed: 18838962. 18838962 PMC9901484

[38] Peng Y, Kang Q, Cheng H, Li X, Sun MH et al. (2003) Transcriptional characterization of bone morphogenetic proteins (BMPs)-mediated osteogenic signaling. J Cell Biochem 90: 1149-1165. doi:10.1002/jcb.10744. PubMed: 14635189. 14635189

[39] He TC, Zhou S, da Costa LT, Yu J, Kinzler KW et al. (1998) A simplified system for generating recombinant adenoviruses. Proc Natl Acad Sci U S A 95: 2509-2514. doi:10.1073/pnas.95.5.2509. PubMed: 9482916. 9482916 PMC19394

[40] Kang Q, Song WX, Luo Q, Tang N, Luo J et al. (2009) A comprehensive analysis of the dual roles of BMPs in regulating adipogenic and osteogenic differentiation of mesenchymal progenitor cells. Stem Cells Dev 18: 545-559. doi:10.1089/scd.2008.0130. PubMed: 18616389. 18616389 PMC3132950

[41] Luo J, Deng ZL, Luo X, Tang N, Song WX et al. (2007) A protocol for rapid generation of recombinant adenoviruses using the AdEasy system. Nat Protoc 2: 1236-1247. doi:10.1038/nprot.2007.135. PubMed: 17546019. 17546019

[42] Peng Y, Kang Q, Luo Q, Jiang W, Si W et al. (2004) Inhibitor of DNA binding/differentiation helix-loop-helix proteins mediate bone morphogenetic protein-induced osteoblast differentiation of mesenchymal stem cells. J Biol Chem 279: 32941-32949. doi:10.1074/jbc.M403344200. PubMed: 15161906. 15161906

[43] Sharff KA, Song WX, Luo X, Tang N, Luo J et al. (2009) Hey1 Basic Helix-Loop-Helix Protein Plays an Important Role in Mediating BMP9-induced Osteogenic Differentiation of Mesenchymal Progenitor Cells. J Biol Chem 284: 649-659. PubMed: 18986983. 18986983 10.1074/jbc.M806389200 PMC2610517

[44] Huang E, Zhu G, Jiang W, Yang K, Gao Y et al. (2012) Growth hormone synergizes with BMP9 in osteogenic differentiation by activating the JAK/STAT/IGF1 pathway in murine multilineage cells. J Bone Miner Res, 27: 1566–75. PubMed: 22467218. 22467218 10.1002/jbmr.1622

[45] Wang Y, Hong S, Li M, Zhang J, Bi Y et al. (2013) Noggin resistance contributes to the potent osteogenic capability of BMP9 in mesenchymal stem cells. J Orthop Res, 31: 1796–803. PubMed: 23861103. 23861103 10.1002/jor.22427

[46] Huang J, Bi Y, Zhu GH, He Y, Su Y et al. (2009) Retinoic acid signalling induces the differentiation of mouse fetal liver-derived hepatic progenitor cells. Liver Int 29: 1569-1581. doi:10.1111/j.1478-3231.2009.02111.x. PubMed: 19737349. 19737349

[47] Zhu GH, Huang J, Bi Y, Su Y, Tang Y et al. (2009) Activation of RXR and RAR signaling promotes myogenic differentiation of myoblastic C2C12 cells. Differentiation 78: 195–204. doi:10.1016/j.diff.2009.06.001. PubMed: 19560855. 19560855 PMC2829657

[48] Rastegar F, Gao JL, Shenaq D, Luo Q, Shi Q et al. (2010) Lysophosphatidic acid acyltransferase beta (LPAATbeta) promotes the tumor growth of human osteosarcoma. PLOS ONE 5: e14182. doi:10.1371/journal.pone.0014182. PubMed: 21152068. 21152068 PMC2995727

[49] Su Y, Wagner ER, Luo Q, Huang J, Chen L et al. (2011) Insulin-like growth factor binding protein 5 suppresses tumor growth and metastasis of human osteosarcoma. Oncogene 30: 3907-3917. doi:10.1038/onc.2011.97. PubMed: 21460855. 21460855

[50] Zhou L, An N, Haydon RC, Zhou Q, Cheng H et al. (2003) Tyrosine kinase inhibitor STI-571/Gleevec down-regulates the beta-catenin signaling activity. Cancer Lett 193: 161-170. doi:10.1016/S0304-3835(03)00013-2. PubMed: 12706873. 12706873 PMC4527752

[51] Zhou L, An N, Jiang W, Haydon R, Cheng H, et al. (2002) Fluorescence-based functional assay for Wnt/beta-catenin signaling activity. Biotechniques 33: 1126-1128, passim 12449394 10.2144/02335dd07

[52] Luo Q, Kang Q, Song WX, Luu HH, Luo X et al. (2007) Selection and validation of optimal siRNA target sites for RNAi-mediated gene silencing. Gene 395: 160-169. doi:10.1016/j.gene.2007.02.030. PubMed: 17449199. 17449199

[53] He BC, Gao JL, Zhang BQ, Luo Q, Shi Q et al. (2011) Tetrandrine inhibits Wnt/beta-catenin signaling and suppresses tumor growth of human colorectal cancer. Mol Pharmacol 79: 211-219. doi:10.1124/mol.110.068668. PubMed: 20978119. 20978119 PMC3033706

[54] He BC, Chen L, Zuo GW, Zhang W, Bi Y et al. (2010) Synergistic antitumor effect of the activated PPARgamma and retinoid receptors on human osteosarcoma. Clin Cancer Res 16: 2235-2245. doi:10.1158/1078-0432.CCR-09-2499. PubMed: 20371684. 20371684

[55] Li M, Chen Y, Bi Y, Jiang W, Luo Q et al. (2013) Establishment and characterization of the reversibly immortalized mouse fetal heart progenitors. Int J Med Sci 10: 1035-1046. doi:10.7150/ijms.6639. PubMed: 23801891. 23801891 PMC3691803

[56] Luo J, Tang M, Huang J, He BC, Gao JL et al. (2010) TGFbeta/BMP type I receptors ALK1 and ALK2 are essential for BMP9-induced osteogenic signaling in mesenchymal stem cells. J Biol Chem 285: 29588-29598. doi:10.1074/jbc.M110.130518. PubMed: 20628059. 20628059 PMC2937990

[57] de Launoit Y, Adamski J (1999) Unique multifunctional HSD17B4 gene product: 17beta-hydroxysteroid dehydrogenase 4 and D-3-hydroxyacyl-coenzyme A dehydrogenase/hydratase involved in Zellweger syndrome. J Mol Endocrinol 22: 227-240. doi:10.1677/jme.0.0220227. PubMed: 10343282. 10343282

[58] Almeida M, Iyer S, Martin-Millan M, Bartell SM, Han L et al. (2013) Estrogen receptor-alpha signaling in osteoblast progenitors stimulates cortical bone accrual. J Clin Invest 123: 394-404. doi:10.1172/JCI65910. PubMed: 23221342. 23221342 PMC3533305

[59] Kouzmenko AP, Takeyama K, Ito S, Furutani T, Sawatsubashi S et al. (2004) Wnt/beta-catenin and estrogen signaling converge in vivo. J Biol Chem 279: 40255-40258. doi:10.1074/jbc.C400331200. PubMed: 15304487. 15304487

[60] Shi B, Liang J, Yang X, Wang Y, Zhao Y et al. (2007) Integration of estrogen and Wnt signaling circuits by the polycomb group protein EZH2 in breast cancer cells. Mol Cell Biol 27: 5105-5119. doi:10.1128/MCB.00162-07. PubMed: 17502350. 17502350 PMC1951944

[61] Armstrong VJ, Muzylak M, Sunters A, Zaman G, Saxon LK et al. (2007) Wnt/beta-catenin signaling is a component of osteoblastic bone cell early responses to load-bearing and requires estrogen receptor alpha. J Biol Chem 282: 20715-20727. doi:10.1074/jbc.M703224200. PubMed: 17491024. 17491024

[62] Liedert A, Wagner L, Seefried L, Ebert R, Jakob F et al. (2010) Estrogen receptor and Wnt signaling interact to regulate early gene expression in response to mechanical strain in osteoblastic cells. Biochem Biophys Res Commun 394: 755-759. doi:10.1016/j.bbrc.2010.03.065. PubMed: 20227388. 20227388

